# Is periodontitis a risk factor for ischemic stroke?: Systematic review and meta-analysis

**DOI:** 10.4317/jced.62538

**Published:** 2025-03-01

**Authors:** Angel Steven Asmat-Abanto, Rosita Elena Espejo-Carrera, Tammy Margarita Honores-Solano, Oscar Martín Del Castillo-Huertas, José Antonio Caballero-Alvarado, Carlos Alberto Minchón-Medina

**Affiliations:** 1 Doctor in Stomatology. Specialist in Periodontics. Professor of the Human Medicine Study Program – Universidad Privada Antenor Orrego, Trujillo, Peru. Professor Coordinator of Periodontology of the Stomatology Study Program - Universidad Privada Antenor Orrego, Trujillo, Peru. Visiting Professor of the Posgraduate School - Universidad Señor de Sipán; 2 Master of Science in Clinical Research. Professor of the Posgraduate School – Universidad Privada Antenor Orrego, Trujillo, Peru. Professor of Periodontology of the Stomatology Study Program - Universidad Privada Antenor Orrego, Trujillo, Peru; 3 Master’s degree in Stomatology. Professor of Stomatology Study Program, Universidad Privada Antenor Orrego, Trujillo, Peru; 4 Doctor in Stomatology. Specialist in Orthodontics and Maxillary Orthopedics. Director of the Stomatology Study Program – Universidad Privada Antenor Orrego, Trujillo, Peru. Professor Coordinator of Orthodontics II of the Stomatology Study Program - Universidad Privada Antenor Orrego, Trujillo, Peru; 5 Doctor in Clinical and Translational Research, Specialist in General Surgery. Professor of Human Medicine Study Program, and Posgraduate School, Universidad Privada Antenor Orrego, Trujillo, Peru. Physician of Surgery Department, Hospital Regional Docente de Trujillo, Peru; 6 Professor of Faculty of Physical Sciences and Mathematics, Department of Statistics, Universidad Nacional de Trujillo, Peru

## Abstract

**Background:**

Evidence suggests an association between periodontitis and ischemic stroke due to the elevated production of inflammatory markers and damage by infectious agents, which would promote a recurrent prothrombotic state. Therefore, the present systematic review and meta-analysis were carried out to determine whether periodontitis is a risk factor for ischemic stroke.

**Material and Methods:**

A systematic search was conducted in five databases, including cohort and case-control studies published up to April 2024, in which periodontitis was evaluated as a risk factor for ischemic stroke through relative risk (RR), hazard ratio (HR) and odds ratio (OR). The Newcastle-Ottawa Scale (NOS) was used to assess the risk of bias, and the GRADE system was used to determine the certainty of the evidence.

**Results:**

Of the 1121 studies found, 16 were included in the qualitative analysis, and 10 were meta-analyzed. The global synthesis showed that periodontitis was a risk factor for ischemic stroke (OR=2.59, I2=96%), with the same result being found for the case-control subgroup (OR=3.44, I²=73%) and the cohort subgroup (OR=2.05, I²=99%). Individuals with periodontitis were also found to be more likely to develop lacunar infarcts (OR=5.00, I2=0%).

**Conclusions:**

Periodontitis is a risk factor for ischemic stroke with very low certainty of evidence and high heterogeneity. Furthermore, individuals with periodontitis were more likely to develop lacunar infarcts, with moderate certainty of evidence and null heterogeneity.

** Key words:**Periodontitis, periodontal diseases, ischemic stroke, lacunar infarction, embolic stroke, thrombotic stroke.

## Introduction

Periodontitis is a chronic inflammation caused by pathogenic microorganisms present in dental biofilm, and leads to the progressive and irreversible destruction of the tooth-supporting tissues (1) It has a global prevalence of between 45% and 90% (1,2), and its most severe form affects between 11.2% and 20% of the population (2,3).

Strokes involve neurological deficit disorders caused by abnormalities in cerebral blood flow (4). They are one of the leading causes of death and long-term disability (5-7) since they cause sensory-motor, musculoskeletal, perceptual, and cognitive sequelae (8) and lead to significant consumption of health resources (9). A stroke may be ischemic if the damage is characterized by occlusion of the cerebral blood vessels or hemorrhagic if it is caused by their rupture (10).

About 80% to 94% of strokes are ischemic (8,11), and their main etiological factor is atherosclerosis of intracranial arteries. However, various risk factors have been identified, such as age, sex, hypertension, diabetes mellitus, smoking, severe tooth loss, hyperlipidemia and obesity (6,11,12). Furthermore, inflammatory markers, such as C-reactive protein (CRP) and interleukin 6 (IL-6), have been associated with the risk of ischemic strokes (6).

Evidence would indicate a possible association between periodontitis and ischemic stroke. Its main mechanisms are indirect damage to vascular function caused by the production of elevated levels of inflammatory markers (5,10) and direct damage caused by infectious agents, such as *Porphyromonas gingivalis*, involved in the pathogenesis of periodontitis. This would promote a prothrombotic state by causing transient and recurrent bacteremia, vascular inflammation, oxidative stress, platelet activation, and coagulation factors, affecting the composition of the thrombus (2,13,14).

Since individual studies might have insufficient power to determine a reliable conclusion, the present systematic review and meta-analysis was performed to evaluate periodontitis as a risk factor for ischemic stroke.

## Material and Methods

-Protocol and registration

The present systematic review was done according to the Preferred Reporting Items for Systematic Reviews and Metanalyses checklist (PRISMA, 2020) (15). The protocol was registered in PROSPERO (CRD42024522139) and INPLASY (INPLASY202440053).

-Focused question

The research question was: Is periodontitis a risk factor for ischemic stroke in adult patients? It was raised according to the PECOS search strategy (population/patients, exposure, comparison, outcomes, and study design), where *P* = adults, E = exposure to periodontitis, C = absence of periodontitis, O = risk of ischemic stroke, and S = cohort and case-control studies.

-Eligibility criteria and process of selection 

Cohort and case-control studies published up to March 2024 that evaluated periodontitis as a risk factor for ischemic stroke in adult patients (over 18 years of age) and calculated the relative risk (RR), hazard ratio (HR), and odds ratio (OR) were included. Studies with incomplete data were excluded.

-Operational definitions

Case-control and cohort studies were included, in which periodontitis was diagnosed by clinical-radiographic evaluation and use of periodontal epidemiological indices. Direct reports and hospital records were also taken into account.

-Search strategy

The search was conducted in PubMed/Medline, Web of Science, Scopus, Embase, and BVS databases in April 2024. In addition, manual searches were performed of the reference lists of all included studies and previously published reviews. The complete search strategy adapted according to the syntax rules of each database is presented in Supplementary document.

-Data extraction and synthesis

All outcome measures that directly assessed the association between periodontitis and ischemic stroke were considered. Two researchers (T.H.S. and R.E.C.) independently selected the articles to be analyzed, first by title and abstract. Then, four researchers performed the full-text analysis in pairs (T.H.S. with R.E.C. and A.A.A. with O.D.C.H). Data were then independently extracted into an Excel spreadsheet (Microsoft® Excel® for Office 365). The articles selected and data extracted were subsequently reviewed and approved by a fifth expert researcher (J.C.A).

-Risk of bias and certainty of evidence

The Newcastle-Ottawa Scale (NOS) tool was used to analyze the risk of bias. Researchers´ disagreements were then resolved. The quality of evidence of the studies included in the meta-analysis was assessed through the GRADE tool, using the GRADEpro GDT software (16).

-Statistical analysis

Data from the studies selected for the meta-analysis of the association between periodontitis and ischemic stroke were evaluated using RevMan software (Review Manager v.5.3, The Cochrane Collaboration), converting effect estimates into ORs. The random effects model was applied to develop the forest plot, and heterogeneity between studies was assessed using the I2 index; sensitivity analysis was also performed to verify each study’s influence on the pooled results. To assess possible publication bias, the Egger test was used, confirmed by the Peters and Harbord tests, using the Stata 16 software (StataCorp LLC, College Station, TX).

## Results

-Selection of studies 

**Figure 1:**
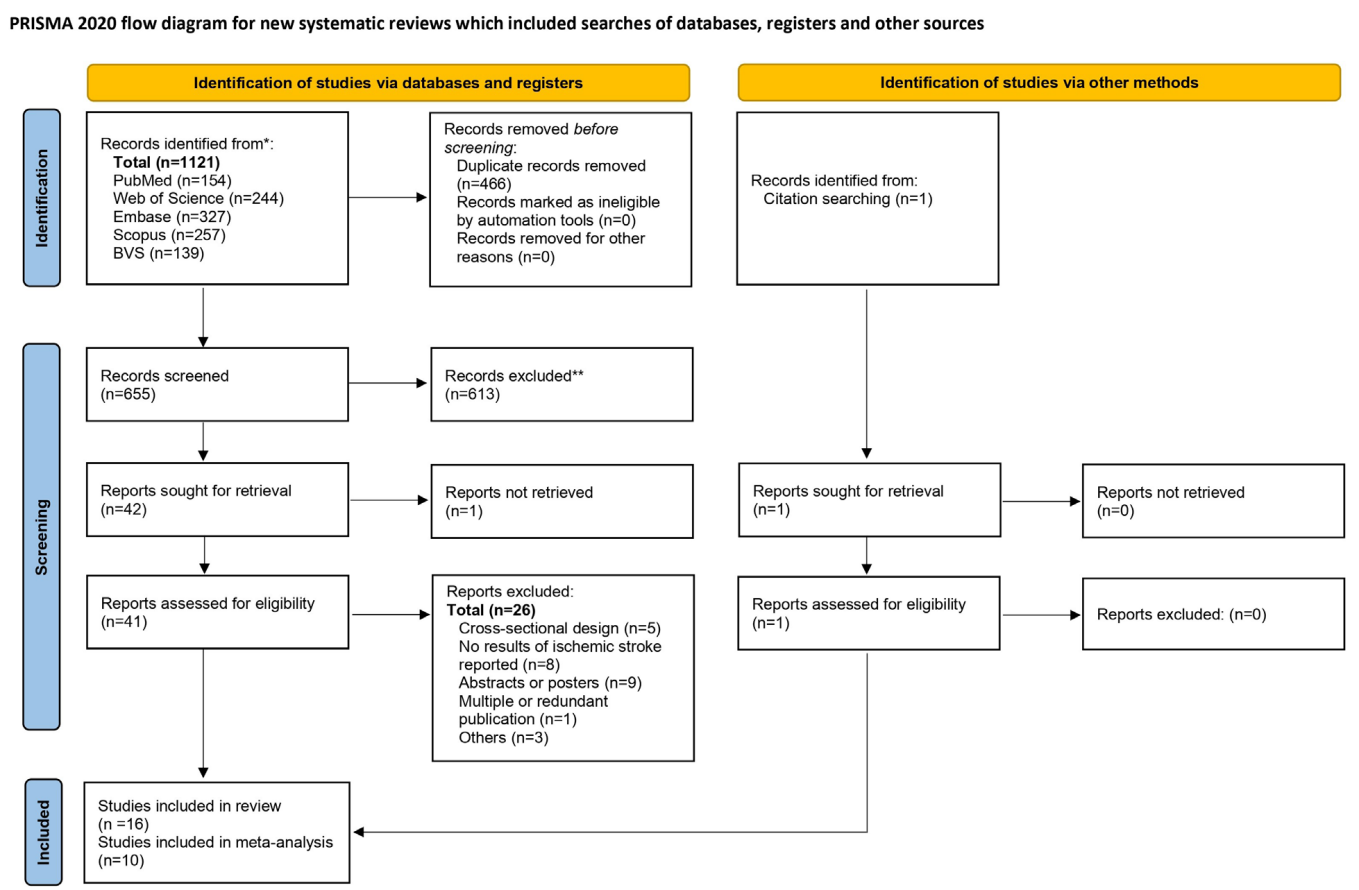
Flow chart for inclusion of the studies evaluating the association between periodontitis and ischemic stroke.

As presented in the PRISMA 2020 flowchart (17) (Fig. 1), 1121 records were retrieved. After removing duplicates and screening by title and abstract, 41 articles remained for full-text evaluation. Of these, 26 were excluded: Eight studies were eliminated because they did not report results of ischemic stroke, nine were abstract/posters, two had periodontitis measurements that did not correspond to the operational definition, five were cross-sectional studies, one compared mild vs severe periodontitis, and one was redundantly published. One study obtained from the references of previous systematic reviews was added, and finally, the qualitative analysis was performed with 16 studies ( Table 1, Table 2), of which 10 were chosen for the meta-analysis.

-Characteristics of the studies

Of the 16 studies, 11 were case-control and 5 cohort studies [2 retrospective cohorts]. The case-control studies evaluated a total of 2917 subjects; the smallest number had 44 patients [22 cases and 22 controls] (18), and the largest number had 771 patients [303 cases and 468 controls] (19), aged between 15 (20) and 80 years of age (21,22). Most studies evaluated ischemic stroke in general; however, three studies included the analysis of ischemic stroke subtypes: transient ischemic attack (TIA) (19) and lacunar infarcts (22,23).

In the cohort studies, 1,864,167 subjects were evaluated; the smallest number had 4364 patients (2527 exposed and 1837 unexposed) (24), and the largest number had 1,584,852 patients (792426 exposed and 792426 unexposed) (25). Of these cohort studies, four evaluated patients diagnosed with periodontitis (exposed) versus patients without periodontitis (unexposed) (24-27), and one study compared patients with periodontitis (exposed) versus patients with gingivitis (unexposed) (28). The ages ranged from 18 (26) to 80 (28). The shortest follow-up period was 14 years (25,28), and the longest was 21 years (27). Of all studies that analyzed ischemic stroke, one included the evaluation of the TIA subtype (25), and one evaluated lacunar, cardioembolic, and thrombotic infarction (24).

The case-control studies analyzed in this review were conducted in Germany (19,29), Spain (22,23), Iran (20,30), Brazil (21), Cuba (18), France (31), India (32) and Korea (33); seven of these studies (20-23,29-31) indicated funding and four (18,19,32,33) did not mention it. Concerning conflicts of interest, seven (20-23,29,31,33) declared no conflict of interest, and four (18,19,30,32) did not declare this item. The cohort studies were conducted in the United States of America (24,27), Taiwan (25,28), and Denmark (26); three of them (24,26,27) indicated funding and two (25,28) did not mention it; while, two (25,28) mentioned that there was no conflict of interest, two (26,27) did not mention it and, the study by Sen (24) indicated in this section that one of its co-authors was an associate editor of the American Academy of Neurology.

-Risk of bias 

Six case-control studies (21-23,30,31,33) presented a high risk of bias, and five (18-20,29,32) had a low risk. Concerning the cohort studies, all five (24-28) presented a low risk of bias ( Table 3).

-Meta-analysis of the synthesis

The results are presented in two meta-analyses, according to the study design and type of ischemic stroke, which included 10 studies and evaluated a total of 882,606 patients with periodontitis and 889,180 patients without periodontitis, considering the OR as a measure of the effect. The global synthesis showed that periodontitis was a risk factor for ischemic stroke (OR=2.59 [95%CI: 1.90; 3.53] and *p*<0.00001) with considerable heterogeneity (I2=96%; *p*<0.00001).

**Figure 2:**
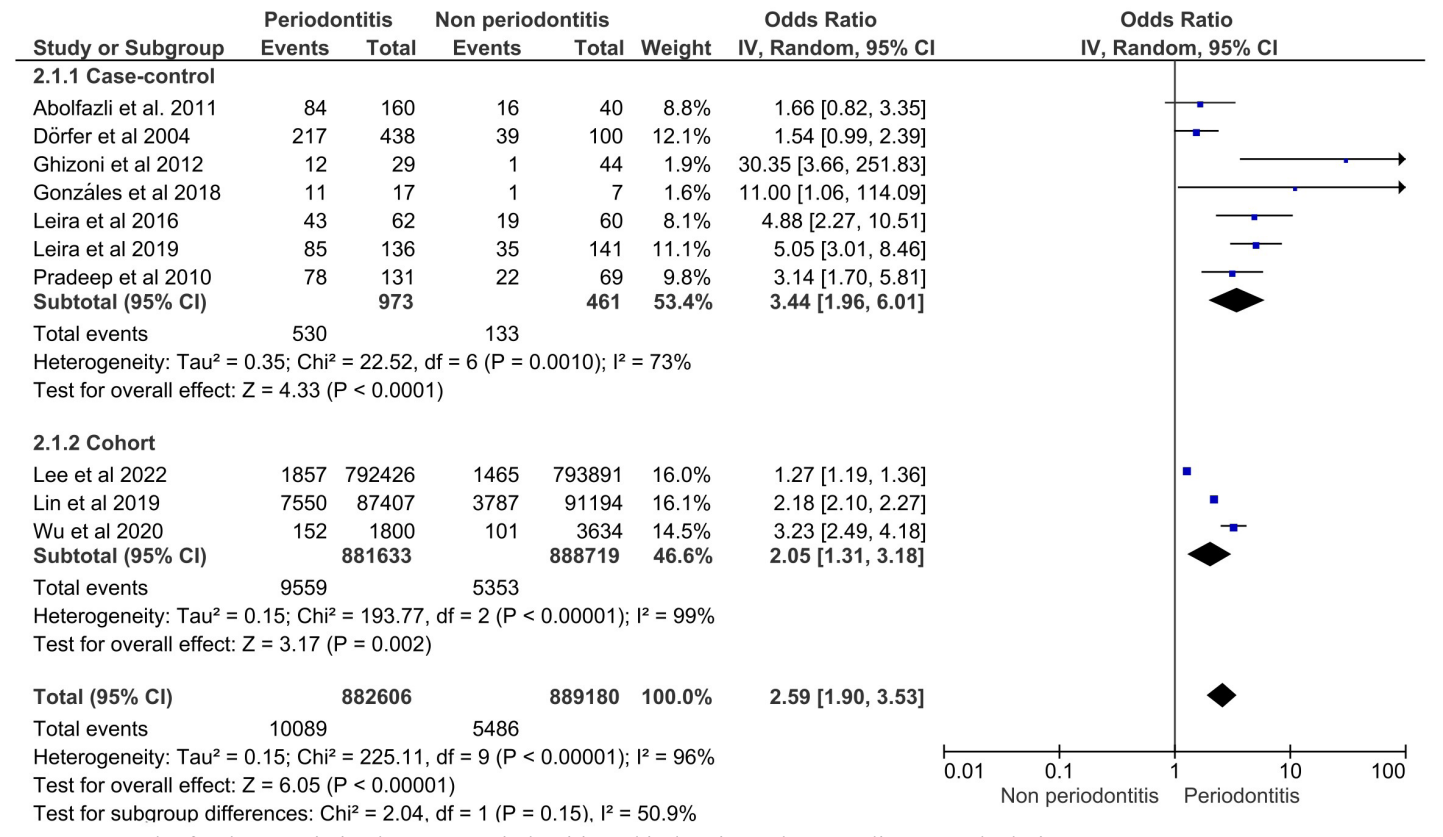
Forest plot for the association between periodontitis and ischemic stroke according to study design.

In the first forest plot (Fig. 2), the subgroup analysis for cases and controls showed an OR=3.44 [95%CI: 1.96; 6.01] and *p*<0.0001, with considerable heterogeneity (I²=73%; *p*=0.0010); similarly, cohort studies obtained an OR=2.05 [95%CI: 1.31; 3.18] and *p*=0.002, with considerable variability (I²=99%; *p*<0.00001) These results confirmed that periodontitis was a risk factor for ischemic stroke for both study designs.

**Figure 3:**
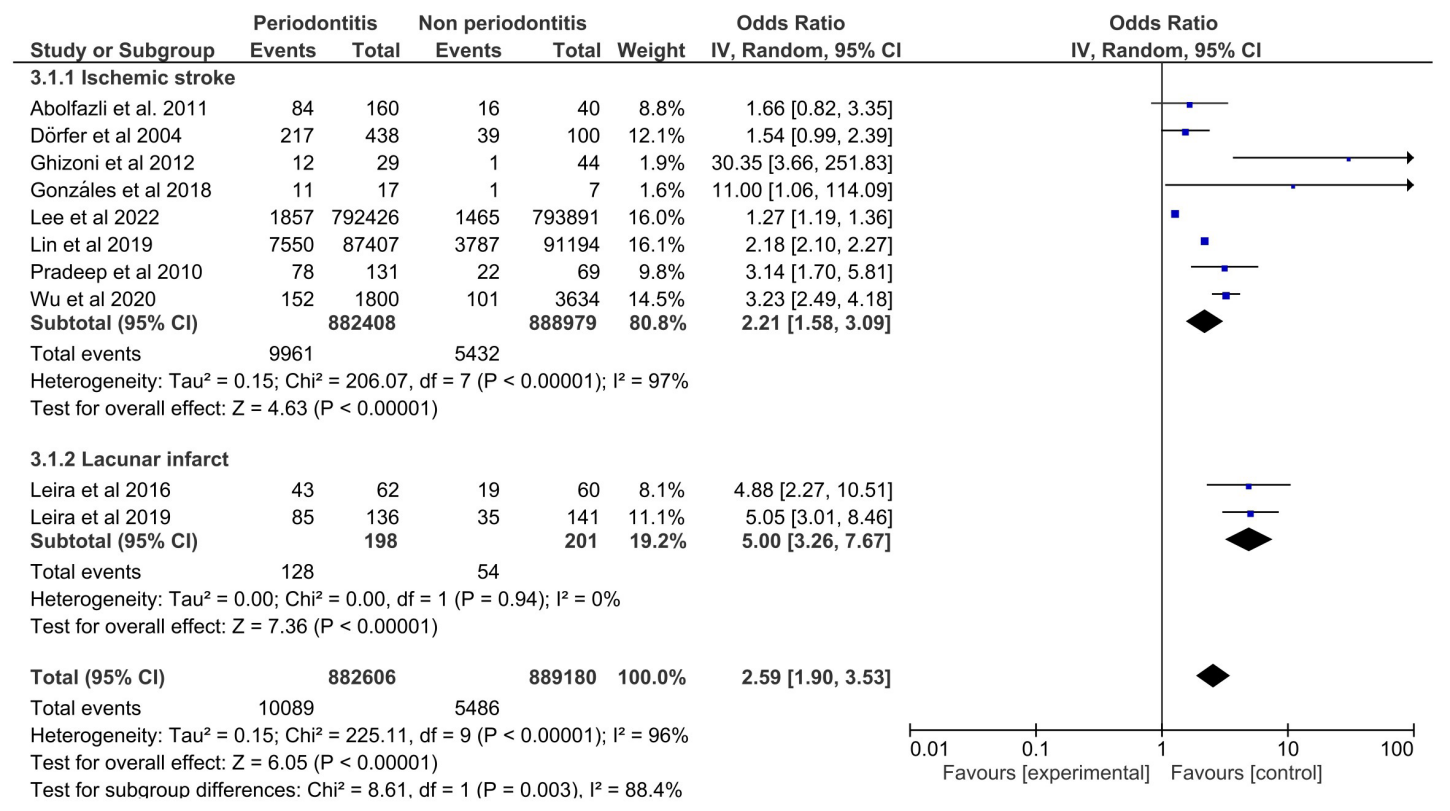
Forest plot for the association between periodontitis and ischemic stroke according to type of stroke.

In the second forest plot (Fig. 3), it was also observed that periodontitis is a risk factor for the ischemic stroke subgroup (OR=2.21 [95%CI: 1.58; 3.09] and *p*<0.00001), with considerable heterogeneity (I2=97%; *p*<0.00001). Similarly, individuals with periodontitis were more likely to develop the lacunar infarct subtype, with moderate OR (OR=5.00 [95%CI: 3.26; 7.67] and *p*<0.00001) and null heterogeneity (I2=0%; *p*=0.94).

-Quality of evidence

**Figure 4:**
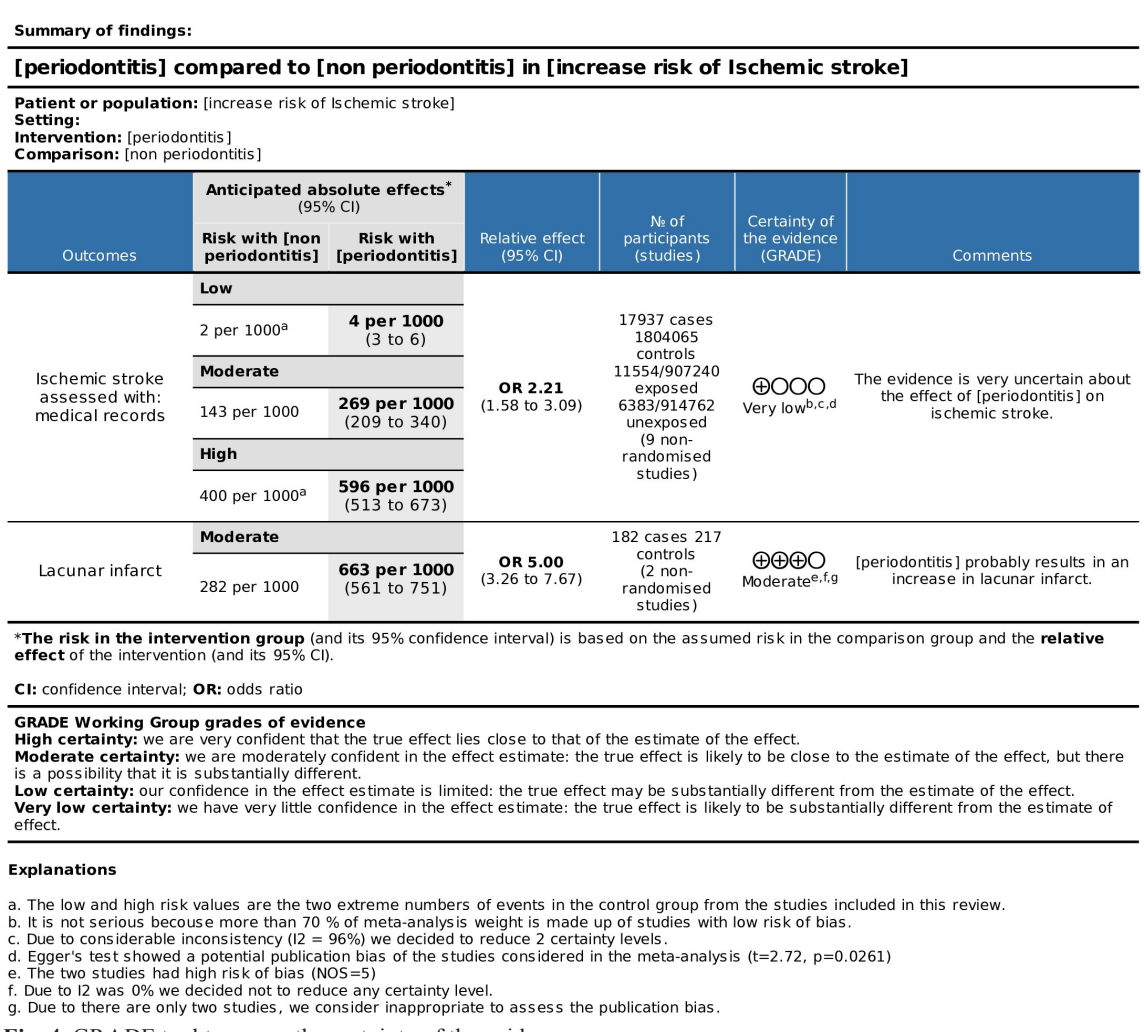
GRADE tool to assess the certainty of the evidence.

The quality of evidence of the studies included in the second meta-analysis, considering the subgroups “ischemic stroke” and “lacunar infarct” as outcomes, was presented in a SoF Table (Fig. 4). Very low certainty of evidence was obtained for periodontitis as a risk factor for ischemic stroke and moderate certainty for periodontitis as a risk factor for lacunar infarct.

-Publication bias and sensitivity analysis 

The Egger test detected a potential publication bias of the studies considered in the meta-analysis (t=2.72, *p*=0.0261), confirmed by Harbord tests (t=2.45, *p*=0.0397). The sensitivity analysis is shown in  Table 4, in which heterogeneity ranged from 96% to 77% (OR = 2.38 to 2.96). Since no significant heterogeneity or effect size changes were evident, it was decided not to exclude any studies.

## Discussion

Thrombosis due to atherosclerotic plaque causes a considerable proportion of ischemic strokes (34). Despite advances in preventive care and treatment, the global incidence and morbidity of ischemic stroke continue to increase. This is explained, among other factors, by the lack of control of vascular risk factors, such as chronic infections and inflammatory diseases (2). In this sense, we can consider as a strength of this review the fact of having studied ischemic stroke, instead of hemorrhagic stroke or stroke in general, because it is the most frequent type of stroke (8,11). There is preliminary evidence that periodontitis increases systemic mediators of inflammation that are risk factors for these atherosclerotic diseases (12).

This systematic review and meta-analysis showed that periodontitis was a risk factor for ischemic stroke, in agreement with Leira *et al*. (35); however, they used RR as a measure of effect in the cohort and case-control subgroups.

According to the meta-analysis of the case-control subgroup, the probability of presenting periodontitis was 3.44 times higher in patients with ischemic stroke compared with those who did not have it. This coincided with the findings of Fagundes *et al*. (10) and Leira *et al*. (35), who used the OR and RR as a measure of effect, respectively.

Similarly, the cohort subgroup showed that there was a higher probability [2.05 times] of developing ischemic stroke in the periodontitis group, coinciding with the reports of Leira *et al*. (35) and Lafon *et al*. (31) who, however, used the RR and estimated adjusted risk (ES) as a measure of effect. Concerning these statistical tests, it should be mentioned that RR and OR are often misused in clinical research. The RR should be restricted exclusively to prospective studies such as cohort studies and not be used in case-control studies (36,37).

Most of the studies analyzed in this review evaluated periodontitis by clinical and/or radiographic diagnosis, considered the standard for periodontal diagnosis (38). Regarding hospital records, despite having been carried out by specialists, could have limitations in the diagnosis of periodontitis. Case-control and cohort studies are observational designs appropriate for evaluating risk factors; however, potential confounders could affect the association’s increase or decrease in these studies.

A relevant finding was that periodontitis is a risk factor for lacunar infarcts with a moderate OR. This is the first systematic review to evaluate this association. The lacunar infarct is a marker of small vessel disease caused by occlusion of perforating intracranial arteries and accounts for up to 25% of ischemic strokes. Periodontitis, mainly in moderate or severe levels, causes a more extensive systemic inflammatory response, thereby promoting dysfunction in the vascular endothelium, with high serum levels of IL-6, PTX3, sTWEAK, and Aβ1-40 associated with a poor prognosis in patients with lacunar, infarcts (39). Only the lacunar infarct subtype was considered for this meta-analysis because studies evaluated other ischemic stroke subtypes, but the number of them was insufficient to be meta-analyzed.

Within the limitations of this meta-analysis, it could be mentioned that the studies by Ghizoni *et al*. (2012) (21) and Gonzales *et al*. (2018) (18) provided much higher OR values than those in the remainder of the studies selected possibly due to the small sample sizes, thereby generating greater imprecision in their results. In this sense, although no significant changes in heterogeneity were evident when applying the sensitivity test, we suggest that they should be considered for exclusion from future meta-analyses.

It is also important to mention that the high heterogeneity of the studies included in the present meta-analysis could be due to differences in the characteristics of the study populations, sample sizes, comparison groups, outcomes, and duration of follow-up periods. Therefore, the findings of this review suggested the need for future studies on this subject, with larger sample sizes and higher methodological quality, to strengthen the evidence on periodontitis as a risk factor for lacunar infarcts. In this way, periodontal control of these patients should be established in the health services to prevent the development of more severe brain lesions.

## Conclusions

Periodontitis is a risk factor for ischemic stroke, with very low certainty of evidence and high heterogeneity. Furthermore, individuals with periodontitis were found to be more likely to develop lacunar infarcts, with moderate certainty of evidence and null heterogeneity. A potential publication bias was detected, making it necessary to conduct future studies on this subject, using larger samples and higher methodological quality, particularly to strengthen the evidence on periodontitis as a risk factor for lacunar infarcts.

## Figures and Tables

**Table 1:** General characteristics of the case-control studies included.

No.	Authors (Country, publication's year)	Age (years)	No. of patients		Distribution by gender		Type of ischemic stroke	Periodontal measurement	Covariables studied	Conclusions
			Cases	Controls	Male	Female				
1	Abolfazli et al. (Iran, 2011)	Cases 54.2 ± 12.15 Controls not hospitalized 52.6 ± 14.28 Hospitalized controls 53.1 ± 12.61	100	100	98	102	IS	Clinical attachment level.	Sex, date of admission, if hospitalized, previous illness, vascular risk factors (including DM, hypertension, VHD, AFib, PAD, obesity), previous IS, smoking, and positive family history of stroke.	PD is associated with the risk of IS.
2	Dörfer et al. (Germany, 2004)	Cases: 59.7 ± 11. Controls: 59.3 ± 8.0	303	300	421	182	IS	Clinical attachment level and radiographic bone loss.	Age, sex, hypertension, smoking, DM, hyperlipidemia, previous stroke or TIA, PAD, CAD, AFib, alcoholism, BMI, family history of stroke, less than 10 years of schooling, educational level, current or past profession, parents' profession, and hot water available at home.	Periodontitis was associated with a risk of IS. Among all periodontal parameters, gingival bleeding appeared to have the strongest association with stroke.
3	Ghizoni et al. (Brazil, 2012)	Cases: 61.08 ± 12.30 Controls: 48±10	13	60	42 (30 controls and 12 IS + HS)	38 (30 controls and 8 IS + HS)	IS	Clinical attachment level and probing depth, and bleeding on probing.	Genetic predisposition, smoking, systemic disorders, hypertension, and CV problems, smoking.	Stroke patients had deeper pockets, more severe attachment loss, more significant bleeding on probing, higher plaque indices, and their pockets harbored higher levels of P. gingivalis. These findings suggested that PD was a risk factor for the development of stroke
4	Gonzáles et al. (Cuba, 2018)	Half of the sample was 71 years old or older	22	22	28	16	AICI	Russell´s Periodontal Index: Codes 6 and 8: periodontitis.	Age, sex, skin color, educational level, job occupation, hypertension, smoking, DM, personal history of stroke, and family history of CVD.	PD predominated over the other risk factors explored for IS, which could suggest an association between the two diseases.
5	Grau et al. (Germany, 2004)	Cases: 59.7±11.2 Community controls: 59.3±8.0 Hospitalized controls 55.3±11.5	303	468	510	261	ISTIA	Clinical attachment level.	Age, sex, smoking, and drinking habits, nutrition, previous stroke or TIA, CAD, PAD, AFib, family history of stroke, educational level, occupation, father's and mother's occupation, BMI, availability of hot water, dental visits, frequency and duration of brushing, previous dental treatments, hypertension, DM, hyperlipidemia, and etiology of cerebral ischemia.	PD was significantly associated with IS.
6	Hashemipour et al. (Iran, 2013)	Cases: 51.89 ± 15.51 Controls: 52.59 ± 17.3	100	100	86	114	IS	Clinical attachment level and probing depth.	Sex, family history of ischemic stroke, DM, PAD, previous stroke, hypertension, AFib, renal failure, and smoking.	There was a significant relationship between stroke and periodontal index.
7	Lafon et al. (France, 2014)	Cases: 60.2 ± 11.8 Controls: 56.1 ± 8.8	48	47	47	48	IS	Probing depth, bleeding on probing, and radiographic bone loss.	Age, sex, educational level, tobacco consumption, alcohol consumption, physical activity, DM, hypercholesterolemia, hypertension, CAD, AFib, PAD, BMI, and hereditary CV disease.	PD may increase the risk of IS due to an inflammatory reaction.
8	Leira et al. (Spain 2016)	Cases: 68.0 (58.0–71.2) Controls: 68.0 (58.0–71.0)	62	60	86	36	LI	Clinical attachment level, probing depth, gingival recession, dental plaque, bleeding on probing, and missing teeth.	Age, sex, smoking habits, alcohol consumption, history of hypertension, DM, hypercholesterolemia, CAD, PAD, and statin use.	Chronic periodontitis was associated with LI with vascular risk factors adjusted.
9	Leira et al. (Spain 2019)	Cases: 66.4 ± 9.9 Controls: 65.4 ± 9.9	120	157	190	87	LI	Clinical attachment level, probing depth, plaque control, bleeding on probing, and missing teeth. Furthermore, periodontal inflamed surface area (PISA) was calculated.	Hypertension, DM, hypercholesterolemia, CAD, PAD, smoking, alcohol consumption, medication (statins, antiplatelet agents, antihypertensives), educational level, leukoaraiosis, carotid atheromatosis, location of LI, last visit to the dentist, frequency of tooth brushing and use of interdental devices.	PD was associated with LI. Moderate to severe active PD was a predictor of worse prognosis in patients with LI.
10	Pradeep et al. (India, 2010)	Cases: 52.3 ± 8.1 Controls: 51.7 ± 9.2	100	100	112	88	IS	Clinical attachment level and probing depth.	Age, sex, dietary history, smoking history, alcohol consumption history, educational level, hypertension, DM, total serum cholesterol level, and family history of stroke.	Periodontitis can also be a risk factor for stroke. Better control of PD can contribute to a decrease in the incidence of strokes.
11	Sim et al. (Korea, 2008)	Cases: 58.87 ± 9.39 Controls: 60.06 ± 11.70	118	214	249	230	IS	Clinical attachment level and probing depth.	Age, sex, income, education, hypertension, DM, CAD, BMI, smoking, alcohol consumption, frequency of daily tooth brushing and annual visit to the dentist and family history of hypertension, DM or heart disease.	Periodontal inflammation was a risk factor for stroke. In patients who had suffered an IS, the association with periodontitis was greater among younger and normotensive adults than in those who had suffered a HS.

Abbreviations: PD = periodontal disease, IS = ischemic stroke, TIA = transient ischemic attack, AICI = atherothrombotic ischemic cerebral infarction, LI = lacunar infarct, TS = thrombotic stroke, DM = diabetes mellitus, AFib = atrial fibrillation, PAD = peripheral arterial disease, CAD = coronary artery disease, VHD = valvular heart disease, BMI = body mass index, CV = cardiovascular, CVD = cerebrovascular disease.

**Table 2:** General characteristics of the cohort studies included.

No.	Authors (Country, publication's year)	Database	Period of follow-up	Age (years)	No. of patients		Type of ischemic stroke	Periodontal measurement	Covariables studied	Conclusions
					Exposed	Unexposed				
1	Hansen et al. (Denmark, 2016)	Danish National Patient Register	15 years	Periodontitis: 57,3 ± 15,1 Controls: 56.6 ± 15.0	Periodontitis: 17691	Without periodontitis 83003	IS	Clinical and radiographic diagnosis of periodontitis.	Alcoholism, cardiac arrhythmia, DM, heart failure, hypertension, CAD/ PAD, kidney disease, and venous thromboembolism.	Periodontitis may be a risk factor for CV disease.
2	Lee et al. (Taiwan, 2022)	The National Health Insurance (NHI) Research Database	14 years	Periodontitis: 37.26 Controls: 37.26	Periodontitis: 792426	Without periodontitis: 792426	TIA	Diagnosis of periodontitis according to ICD-9-CM codes: 523.3 Aggressive and acute periodontitis 523.4 Chronic periodontitis 523.5 Periodontosis.	Age, sex, hypertension, DM, CAD, congestive heart failure, AFib, lipoid metabolism disorders.	In young adults, periodontitis was a potential risk factor for TIA/IS minor.
3	Lin et al. (Taiwan, 2019)	The Taiwan National Health Insurance Research Database	14 years	Periodontitis: 46.7±14.3 Controls: 38±13.9	Periodontitis: 87407	Gingivitis: 74516	IS	Diagnosis of periodontitis according to ICD-9-CM codes: 523.3 Aggressive and acute periodontitis 523.4 Chronic periodontitis 523.5 Periodontosis.	Age, sex, dental treatment groups, and initial comorbidities (Hypertension, DM, COPD, depressive disorder, anxiety, insomnia, sleep problems and chronic kidney disease)	Periodontitis was a risk factor for IS in comparison with patients with gingivitis. Both dental scaling and intensive treatment of periodontal disease were associated with a lower risk of IS.
4	Sen et al. (USA, 2018)	ARIC (Atherosclerosis Risk in Communities)	15 years (median)	Mild PD= 61,7 ± 5,5Posterior PD = 62,8± 5,6 Severe PD = 61,8± 5,7 Controls: 61.7 ± 5.5	Mild PD = 1036 Posterior PD = 993 Severe PD = 498	Healthy = 1837	ISLI CES TS	Clinical attachment level, probing depth, bleeding on probing.	Age, sex, race, BMI, waist-hip ratio, lipid profile, hypertension, DM, educational level, smoking, alcohol.	PD was a risk factor for the incidence of IS.
5	Wu et al. (USA, 2000)	First National Health and Nutrition Examination Survey (NHANES I)	21 years	48.31 ± 15.77	Periodontitis: 1800	No disease: 3634	IS	Gingival inflammation, presence or absence of periodontal pocket, and tooth mobility.	Age, race, sex, years of education, family income level, smoking, DM, alcohol consumption, total serum cholesterol levels and BMI.	Periodontitis was associated with an increased risk of stroke, particularly non-hemorrhagic stroke.

Abbreviations: IS = ischemic stroke, TIA = transient ischemic attack, LI = lacunar infarct, TS = thrombotic stroke, DM = diabetes mellitus, AFib = atrial fibrillation, PAD = peripheral arterial disease, CAD = coronary artery disease, BMI = body mass index, COPD = chronic obstructive pulmonary disease, CES = cardioembolic stroke.

**Table 3:** Summary of the risk of bias assessment for case-control and cohort studies – New Castle Ottawa.

CASE-CONTROL STUDIES									
Study	Selection				Comparability	Exposure			NOS
Is the case definition adequate?	Representativeness of the cases	Selection of controls	Definition of controls	Comparability of cases and controls based on the design or analysis	Ascertainment of exposure	The same method of ascertainment for cases and controls	Non-response rate
Abolfazli et al.	*	*	*	-	**	-	*	*	7
Dörfer et al.	*	*	*	*	**	-	*	*	8
Ghizoni et al.	*	*	*	-	**	-	*	-	6
Gonzáles et al.	*	*	-	*	**	-	*	*	7
Grau et al.	*	*	*	*	**	-	*	*	8
Lafon et al. 2014	*	*	-	*	-	-	*	-	4
Leira et al. 2016	*	*	-	-	**	-	*	-	5
Leira et al. 2019	*	*	-	-	**	-	*	-	5
Pradeep et al.	*	*	-	*	**	-	*	*	7
Sim et al.	*	*	*	-	*	-	*	*	6
Hashemipour et al.	*	*	-	-	**	-	*	*	6
COHORT STUDIES									
Study	Selection				Comparability	Outcome			Nos
	Representativeness of the exposed cohort	Selection of the non-exposed cohort	Ascertainment of exposure	Demonstration that outcome of interest was not present at start of the study	Comparability of cohorts based on the design or analysis	Assessment of outcome	Was follow-up long enough for outcomes to occur?	Adequacy of follow-up of cohorts	
Lee et al.	*	*	*	*	**	*	*	*	9
Lin et al.	*	*	*	*	**	*	*	*	9
Hansen et al.	*	*	*	*	**	*	*	-	8
Sen et al.	*	*	*	*	**	*	*	*	9
Wu et al.	*	*	*	*	**	*	*	*	9

**Table 4:** Sensitivity analysis.

Studies	Number of studies	Heterogenity		Model	Meta-analysis		
		I^2^(%)	p		OR	CI 95%	p
Abolfazli et al. (2011)	9	96	< 0.00001	Random-effects	2.71	1.96-3.75	< 0.00001
Dörfer et al. (2004)	9	96	< 0.00001	Random-effects	2.79	2.00-3.90	< 0.00001
Ghizoni et al. (2012)	9	96	< 0.00001	Random-effects	2.47	1.81-3.37	< 0.00001
Gonzáles et al. (2018)	9	96	< 0.00001	Random-effects	2.53	1.86-3.46	< 0.00001
Lee et al. (2022)	9	77	< 0.0001	Random-effects	2.92	2.15-3.97	< 0.00001
Leira et al. (2016)	9	96	< 0.00001	Random-effects	2.45	1.78-3.38	< 0.00001
Leira et al. (2019)	9	96	< 0.00001	Random-effects	2.38	1.72-3.28	< 0.00001
Lin et al. (2019)	9	92	< 0.00001	Random-effects	2.96	1.80-4.89	< 0.0001
Pradeep et al. (2010)	9	96	< 0.00001	Random-effects	2.54	1.83-3.52	< 0.00001
Wu et al. (2020)	9	96	< 0.00001	Random-effects	2.50	1.79-3.49	< 0.00001

## Data Availability

The datasets used and/or analyzed during the current study are available from the corresponding author.
